# Changes in health-related quality of life among older adults aging with long-term spinal cord injury

**DOI:** 10.1038/s41393-020-00579-0

**Published:** 2020-11-12

**Authors:** Sophie Jörgensen, Maria Valentina Costa Andersson, Jan Lexell

**Affiliations:** 1 Department of Health Sciences, Lund University, Lund, Sweden; 2 Department of Rehabilitation Medicine, Skåne University Hospital, Lund, Sweden

**Keywords:** Spinal cord diseases, Quality of life

## Abstract

**Study design:**

Cross-sectional and longitudinal.

**Objectives:**

To (i) describe health-related quality of life (HRQoL) and changes over 6 years in older adults aging with long-term spinal cord injury (SCI) and (ii) investigate how changes in HRQoL are associated with age, gender, and injury characteristics.

**Setting:**

Community in southern Sweden.

**Methods:**

From the initial 123 participants (years 2011–2012) in the Swedish Aging with Spinal Cord Injury Study (SASCIS), 77 individuals (32% women, C1-L3, AIS A–D, median age 66 years, median time since injury 31 years, 30% complete injuries) were assessed 6 years later. HRQoL was rated with the Spinal Cord Injury Quality of Life Questionnaire (SCI QL-23). Associations were investigated using multivariable linear regression analyses.

**Results:**

The median rating of global QoL (scale range 0–100) was relatively high at both assessments (67 and 83, respectively). There was a large variability in all HRQoL-domains and no significant changes over 6 years. As compared to an AIS D injury, a tetraplegia AIS A–C injury and tetraplegia and paraplegia AIS A–C injuries were associated with positive change in depressive symptoms and global QoL, respectively.

**Conclusions:**

Older adults aging with long-term SCI show large variations in all HRQoL-domains and have the potential to maintain a high and stable level of HRQoL over time. Persons with AIS D injuries may need increased attention to mitigate negative changes in depressive symptoms and global QoL. Further studies are needed to identify modifiable factors associated with changes in HRQoL in older adults aging with long-term SCI.

## Introduction

An overall purpose of rehabilitation and long-term management after a spinal cord injury (SCI) is to enable individuals to reach and maintain the highest possible level of quality of life (QoL) [1] throughout their lifespan. Despite the growing attention to research and clinical practice regarding aging with long-term SCI, knowledge of QoL many years after a SCI is still very limited.

QoL is a broad concept and commonly referred to as health-related QoL (HRQoL), which can be defined as “components of overall (objective) QoL that center upon or are directly or indirectly affected by health, disease, disorder and injury […] [2]”. The Spinal Cord Injury Quality of Life Questionnaire (SCI QL-23) [3] is a measure of HRQoL developed specifically for people with SCI. It considers the physical, psychological, and social burden of SCI and has been validated for Swedish conditions [4]. Using the SCI QL-23, Elfström et al. [4] found that the use of appropriate coping strategies with higher acceptance and lower social reliance contribute to a greater HRQoL. Similar results were found in a recent systematic review where acceptance coping was associated with higher levels of QoL [5]. However, conflicting results have been found how different domains of HRQoL are associated with age, gender and injury characteristics [6–9]. These associations have mostly been studied cross-sectionally and in samples including younger persons with a wide range of years since injury.

As many persons with SCI today live into old age with long-term injury, knowledge is needed on how the aging process influences HRQoL. There are some longitudinal studies describing various aspects of QoL [10–12]. They often report on an improvement in QoL after the acute injury, which then remains high and stable over time [10, 13, 14]. However, satisfaction with health seems to decline quite rapidly 30–40 years after injury among persons who have lived more than 40 years with SCI [15]. To the best of our knowledge, no study has focused on SCI-specific HRQoL and changes over time in a population of older adults with long-term SCI. Moreover, longitudinal data on aging with SCI from a Northern European perspective (i.e., the Nordic countries and the Baltic states) are non existent. An increased understanding of HRQoL, changes over time and associated factors as one ages with long-term SCI can facilitate the design of rehabilitation interventions and follow-up programs to support healthy aging after SCI.

The aims of this study are (i) to describe HRQoL and changes over 6 years in older adults aging with long-term SCI and (ii) to investigate how changes in HRQoL are associated with age, gender, and injury characteristics. Based on previous research [4, 6, 9, 10, 13, 14] and clinical experience, we hypothesize that HRQoL remains relatively stable over time, but that domains covering SCI-related disability decline, and that injury characteristics are stronger explanatory factors for changes in HRQoL than age and gender.

## Methods

### Research design

As part of the large longitudinal, population-based cohort study the Swedish Aging with Spinal Cord Injury Study (SASCIS) [16], the present study is based on data collected at the initial assessment (years 2011–2012) and at the second assessment after on average 6 years (years 2017–2018). The SASCIS is the first longitudinal study on aging with SCI conducted from a Northern European perspective and aims to provide knowledge on factors associated with healthy aging in people with long-term SCI [16]. We aim to perform follow-up assessments with 6 year intervals. The results from our previous cross-sectional studies are overall positive, showing that these older adults with long-term SCI exhibit a relatively high level of physical independence, are generally satisfied with their lives, have a strong sense of coherence and a low presence of probable depression. However, our results also provide indications for a proactive approach in clinical follow-up, especially regarding pain, depressive symptoms, satisfaction with sexual life, and lifestyle-related factors [17–21].

Data were collected through structured interviews and assessments during home visits, using 12 (eight generic and four SCI-specific) widely used and internationally validated assessment tools together with a study-specific questionnaire (for details, see Jörgensen et al. [16]), focusing on different aspects of functioning, disability, and health. All assessment tools initially included were used at the second assessment to enable comparisons over time. In the present study, a subset of the data was used to address the aims. The SASCIS follows the STROBE (Strengthening the reporting of observational studies in epidemiology) recommendations on reporting of cohort studies [22].

### Participants

Participants in the SASCIS were residing in the community and recruited from databases at the SCI Unit at Skåne University Hospital in Lund, Sweden. The SCI Unit provides primary rehabilitation to persons with newly acquired SCI, covering a catchment area of 1.9 million people in southern Sweden. The two main inclusion criteria of the SASCIS were: (i) aged 50 years or older and (ii) at least 10 years after a traumatic or an acquired, nonprogressive, nontraumatic SCI. In addition, participants were required to understand Swedish and reside in the southern part of Sweden.

### Data collection

#### Gender, age, and injury characteristics

Data on gender, age, and injury characteristics (age at injury, time since injury, and cause of injury (dichotomized as traumatic/non traumatic) and level and severity of injury (according to the International Standards for Neurological Classification of Spinal Cord Injury (ISNCSCI) [23]) were collected through a study-specific questionnaire and retrieved from the initial data collection.

#### Health-related quality of life

Data on HRQoL were collected using the Spinal Cord Injury Quality of Life Questionnaire (SCI QL-23) [3]. The SCI QL-23 is a 23-item questionnaire developed in Sweden through a combination of items from the Sickness Impact Profile (SIP) and the Hospital Anxiety and Depression scale (HAD) with questions regarding SCI-related problems. It rates HRQoL across four main domains: (i) functioning (ten items assessing physical and social limitations), (ii) depressive feelings (six items assessing distress and depressive symptoms), (iii) perception of SCI-related problems (six items assessing perception of loss of independence, of SCI-related complications and of social stigma resulting from injury-related problems) and (iv) global QoL (one item to rate the overall QoL on a scale from one to seven). Scores from each domain are transformed into a 0–100 scale. Lower scores on functioning, depressive feelings and SCI-related problems indicate greater SCI-related QoL, whereas higher scores on the global QoL item indicate greater overall QoL. Content validity of the instrument is assured by the combination of both generic and SCI-specific aspects of physical and psychosocial functioning and well-being along with overall QoL [6]. Internal consistency has been reported as high (Cronbach’s alpha ≥0.85) and the tool is sensitive to clinically relevant differences regarding level and severity of injury [6].

### Statistical methods

Data analyses were performed using the Statistical Package for Social Sciences Software version 25 (IBM Corporation, Armonk, NY, USA). Data on age, gender, injury characteristics, and HRQoL are presented using percentages (%) and median (25th–75th percentiles; Tukey’s hinges). To investigate associations between HRQoL domains at the second assessment and age, gender and injury characteristics, the Mann–Whitney *U* test/the Kruskal–Wallis test and Spearman’s rho were used: For categorical variables with two groups (gender, cause of injury), differences between subgroups were analysed using the Mann–Whitney *U* test and data are presented as median, effect size, and *p* value. For categorical variables with three groups (level and severity of injury), differences between subgroups were analysed using the Kruskal–Wallis test and data are presented as median and *p* value with post hoc analyses in footnote. For continuous variables (age, age at time of injury, and time since injury), correlations were investigated using Spearman’s rho and data are presented as Spearman’s rho; *p* value. Changes from the initial to the second assessment in each domain of HRQoL were assessed using the Wilcoxon signed-rank test. Effect sizes were calculated as *r* = *Z* value/square root of *n* (*n* = total number of observations) [24].

To investigate associations between changes in HRQoL and age, gender and injury characteristics, multivariable linear regression analyses were computed. For these analyses, a change score was calculated where the scores for the four domains in the SCI QL-23 at the initial assessment were subtracted from the scores at the second assessment. Two outliers (mean ± 3 SD) were detected in the change score of the functioning domain and one outlier was detected in the change score of the depressive feelings domain; the outliers were reduced to the next lowest value in the distribution. A sensitivity analysis did not reveal any differences between using untransformed and transformed scores. The change score was used as the dependent variable and independent variables were gender, age at the second assessment, time since injury at the second assessment, level and severity of injury (with all AIS D as reference category), and cause of injury. Models were controlled for the score of the investigated domain in the SCI QL-23 at the initial assessment. Only significant models are presented. The presented models exhibited no residual outliers, no influential cases, no multicollinearity, no heteroscedasticity and the residuals were normally distributed. Adjusted *R*^2^ is used as a measure of explained variance. The relationship between significant independent variables and the dependent variable were further investigated using descriptive statistics. A *p* value < 0.05 was considered statistically significant.

## Results

### Participants

**Fig. 1:**
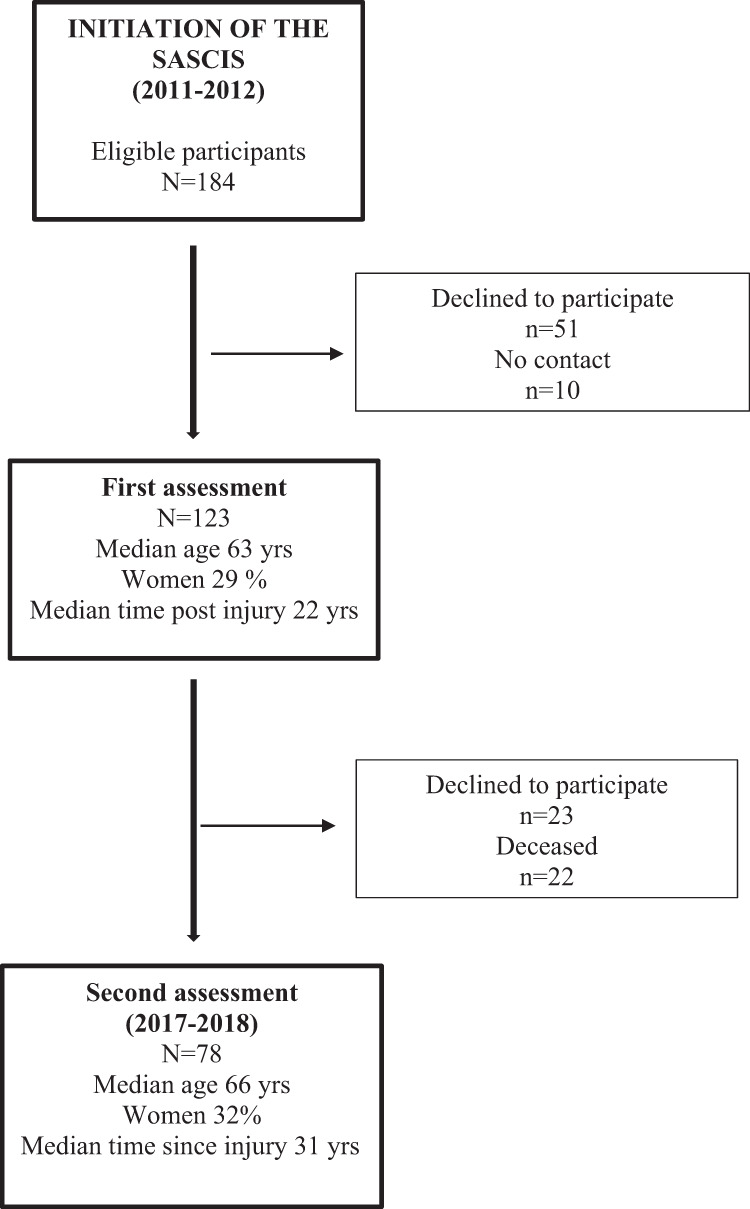
Describes the recrutiment procedure of the Swedish Aging with Spinal Cord Injury Study (SASCIS) from the initiation in 2011–2012 to the second assessment in 2017–2018. The boxes to the left present the number and characteristics of the final participants and the boxes to the right present the non-participants.

A flow chart of the recruitment process for the initial and second assessments is presented in Fig. 1. Of 184 eligible participants, the final study sample at the initial assessment (years 2011–2012) included 123 participants. At the time of the second assessment (years 2017–2018), 101 people from the original sample were alive, and 23 declined to participate further. When comparing the deceased (*n* = 22) with the potential participants (*n* = 101), they were older at baseline (*p* = 0.006, median age 66 years and 61 years, respectively). In addition, more of the initial participants with tetraplegia AIS A–C injuries were deceased compared to those with paraplegia AIS A–C and AIS D injuries (*p* = 0.044; 36% as compared to 15% and 13%, respectively).

The final study sample at the second assessment comprised 78 participants. Based on the ISNCSCI [23], three different groups of SCI level and severity were formed: (i) tetraplegia AIS A–C (*n* = 12), (ii) paraplegia AIS A–C (*n* = 26), and (iii) all AIS D (*n* = 40). When comparing the final study sample at the initial assessment (*n* = 78) with the dropouts (*n* = 23) no significant differences were found with regard to gender, age, level of injury (tetraplegia versus paraplegia), cause (traumatic versus nontraumatic), or severity of injury (complete versus incomplete). The participants at the second assessment were younger at the time of injury (*p* = 0.021, median 34.5 years and 50 years, respectively) and had lived longer with their injury (*p* = 0.014, median 25 years and 15 years, respectively) as compared to the non participants.

### Age, gender and injury characteristics

From the original SASCIS study sample (*n* = 123), 77 participants had completed the SCI QL-23 at the second assessment and were included in the present study. Data on their age, gender, and injury characteristics are presented in Table 1.

**Table 1:** Age, gender, and injury characteristics of older adults aging with long-term spinal cord injury.

	*n* (%)/median (25th–75th percentiles)
Age (years)	66 (62–73)
Gender	
Men	52 (68)
Women	25 (32)
Age at time of injury (years)	35 (22–51)
Time since injury (years)	31 (20–39)
Cause of injury	
Traumatic^a^	49 (64)
Non-traumatic^b^	28 (36)
Level and severity of injury	
Tetraplegia AIS A–C	12 (16)
Paraplegia AIS A–C	25 (32)
All AIS D	40 (52)

*AIS* American Spinal Injury Association Impairment Scale [23].

^a^Traffic/transportation, fall, workplace accident, diving accident, gunshot/assault/torture, other traumatic.

^b^Spinal tumor, spinal disk herniation, spinal arteriovenous malformation, spinal infarction, spinal infection.

### Health-related quality of life among older adults aging with long-term spinal cord injury and changes over 6 years

Data on the four domains of the SCI QL-23 and changes in HRQoL from the initial to the second assessment are presented in Table 2. At group level, there were no significant changes over time in any of the four domains of the SCI QL-23.

**Table 2:** Health-related quality of life among older adults aging with long-term spinal cord injury at the initial and the second assessment.

SCI QL-23 (scale 0–100)	Initial assessment median (25th–75th percentiles)	Second assessment median (25th–75th percentiles)	Effect size; *P* value^a^
Physical/social functioning	29 (12–49)	34 (19–56)	0.12; 0.13
Depressive feelings	11 (6–22)	11 (5.5–22)	0.04; 0.65
Injury-related problems	44 (28–64)	44 (28–67)	0.12; 0.14
Global QoL	67 (50–83)	83 (67–92)	0.11; 0.18

SCI QL-23, Spinal Cord Injury Quality of Life Questionnaire: lower scores on functioning, depressive feelings and injury-related problems indicate greater QoL. Higher scores on global QoL indicate greater QoL.

^a^Differences between the initial and the second assessment were analysed with the Wilcoxon signed-rank test. Effect sizes were calculated as *r* = *Z*-value/square root of the total number of observations.

### Associations between health-related quality of life and age, gender, and injury characteristics

Associations between HRQoL at the second assessment and age, gender and injury characteristics are presented in Table 3. Participants with traumatic SCI rated significantly poorer on functioning (*r* = 0.24; *p* = 0.037) and injury-related problems (*r* = 0.31; *p* = 0.007) compared to participants with nontraumatic SCI. Participants with a tetraplegia AIS A–C injury rated significantly poorer on functioning compared to participants with an AIS D injury (*r* = 0.52; *p* = 0.004).

**Table 3:** Associations between health-related quality of life and age, gender, and injury characteristics among older adults aging with long-term spinal cord injury at the second assessment.

	SCI QL-23 (scale 0–100)							
	Functioning		Depressive feelings		Injury-related problems		Global quality of life	
	*r*_s_	*p* value	*r*_s_	*p* value	*r*_s_	*p* value	*r*_s_	*p* value
Age (years)	0.15	0.19	−0.004	0.97	0.06	0.61	0.074	0.52
Age at time of injury (years)	0.047	0.68	0.036	0.76	0.054	0.64	0.13	0.26
Time since injury (years)	0.11	0.36	−0.03	0.81	0.02	0.87	−0.14	0.22

	Median	ES; *p* value^a^	Median	ES; *p* value^a^	Median	ES; *p* value^a^	Median	ES; *p* value^a^
Traumatic/non-traumatic	36/31	0.24; 0.037*	11/9	0.12; 0.29	56/33	0.31; 0.007*	83/83	0.02; 0.856
Men/women	34/39	0.05; 0.68	6/17	0.16; 0.15	44/39	0.007; 0.95	83/83	0.04; 0.72

	Median	*p* value^b^	Median	*p* value^b^	Median	*p* value^b^	Median	*p* value^b^
Tetra AIS A–C/Para AIS A–C/All AIS D	58/35/30	0.003*^c^	6/11/11	0.11	39/59/42	0.55	83/83/67	0.039*^d^

*SCI QL-23* Spinal Cord Injury Quality of Life Questionnaire, *r*_s_ Spearman’s rho, *ES* effect size: lower scores on functioning, depressive feelings and injury-related problems indicate greater quality of life (*QoL*). Higher scores on global QoL indicate greater QoL.

Effect sizes were calculated as *r* = *Z*-value/square root of the total number of observations.

*Statistically significant result (*p* < 0.05).

^a^Analysed with the Mann–Whitney *U* test.

^b^Analysed with the Kruskal–Wallis test.

^c^Post hoc analyses with Bonferroni correction revealed significant differences between tetra AIS A–C and all AIS D, *r* = 0.52; *p* = 0.004.

^d^Post hoc analyses with Bonferroni correction revealed no significant differences between groups.

### Associations between changes in health-related quality of life over 6 years and age, gender, and injury characteristics

Associations between changes in HRQoL from the initial to the second assessment and age, gender, and injury characteristics are presented in Table 4. The models with changes in functioning and perception of SCI-related problems as dependent variables did not reach significance. For depressive feelings, the independent variables explained 16% of the variance. Regardless of the rating at the initial assessment, a tetraplegia AIS A–C injury was significantly associated with a negative change (i.e., fewer depressive feelings) as compared to an AIS D injury over time (mean change score −6.9 and 1.65, respectively). For global QoL, the independent variables explained 43% of the variance. Regardless of the rating at the initial assessment, tetraplegia and paraplegia AIS A–C injuries were significantly associated with positive change (i.e., greater global QoL) as compared to an AIS D injury over time (mean change score 16.3, 9.0, and −0.83, respectively).

**Table 4:** Multivariable linear regression models investigating the association between changes in health-related quality of life and age, gender, and injury characteristics among older adults aging with long-term spinal cord injury.

Independent variables	Depressive feelings^a^			Global quality of life^a^		
	Beta	*B*	95% CI for *B*	Beta	*B*	95% CI for *B*
Age at second assessment (years)	−0.13	−0.25	−0.70, 0.20	0.31	0.10	−0.31, 0.92
Female gender	0.07	2.32	−5.26, 9.90	−0.09	−0.72	−10.93, 9.49
Time since injury at second assessment (years)	−0.07	−0.05	−0.41, 0.27	−0.16	−0.39	−0.86, 0.09
Non-traumatic injury	−0.14	−4.43	−13.51, 4.65	0.05	2.62	−9.92, 15.16
Tetraplegia AIS A–C^b^	−0.29*	−12.16*	−22.39, −1.94	0.36**	25.35**	11.15, 39.54
Paraplegia AIS A–C^b^	−0.14	−4.55	−13.26, 4.16	0.31**	16.79**	4.48, 29.09
Score at initial assessment	−0.47**	−0.46**	−0.68, −0.24	−0.67**	−0.76**	−0.96, 15.16
Adjusted *R*^2^	0.16			0.43		

*Beta* standardized regression coefficient, *B* unstandardized regression coefficient, *CI* confidence interval

*p  <  0.05; **p < 0.01.

^a^Change scores (scores at the initial assessment were subtracted from the scores at the second assessment) for the subscales Depressive feelings and Global quality of life in the Spinal Cord Injury Quality of Life Questionnaire (SCI QL-23). Lower scores on depressive feelings indicate greater quality of life (QoL). Higher scores on global QoL indicate greater QoL.

^b^Reference category: all AIS D.

## Discussion

The present study describes, for the first time, HRQoL and changes over time among older adults aging with long-term SCI in a Northern European perspective. The median rating of global QoL was relatively high at both assessments (67 and 83, respectively), and all HRQoL-domains exhibited large variability. Our hypotheses were partly supported as HRQoL remained stable over 6 years. Moreover, the level and severity of injury explained some variance in depressive feelings and global QoL, where a higher level and more severe SCI was associated with better outcomes.

### Health-related quality of life among older adults aging with long-term spinal cord injury and changes over 6 years

The participants rated very similarly across the four domains of HRQoL at both assessments, indicating a stable situation over time. In comparison with a previous Swedish study (*n* = 256, median age 40 years, median time since injury 7 years) [4], our participants rated a greater global QoL. These positive findings were expected as previous studies have found that global QoL reaches a relatively high and stable level many years after SCI [10, 13, 14]. In the SASCIS sample, we have previously reported on relatively high levels of global life satisfaction [17, 20]. Although life satisfaction and HRQoL are not identical constructs, it could be assumed that a person’s overall satisfaction with their life situation would relate to the rating of global QoL. The findings may reflect that those who are better adjusted and more satisfied with their life situation take part in follow-ups [12]. Another potential explanation is that priorities and expectations have shifted after the injury and with age [14]. Taken together, the present and previous findings show that despite aging with a lifelong disability, persons with SCI have the potential to reach and maintain a relatively high global QoL over time and many years after the injury.

When assessing specific domains of HRQoL, a slightly different picture emerged. As compared to other Swedish samples [4], our participants rated similarly or poorer on physical and social functioning, somewhat better on perceptions of injury-related problems and much better on depressive feelings. These results indicate that our participants experienced a certain degree of physical and social limitations, loss of independence and difficulties regarding injury-related problems, but still had few signs of distress and depression. Again, this was not unexpected as we have previously found a low (5%) occurrence of probable depression in the SASCIS sample [19]. These findings therefore contradict the common notion that older adults in general [25], and people with SCI in particular [26] are at high risk of depressive disorders.

Previous studies have found a longer time since injury to be associated with greater global QoL [7] and to be a protective factor against depression [27]. At the second assessment, the SASCIS participants had lived with SCI for a median time of 31 years, which could provide one explanation to the overall positive results. Another potential explanation is that SCI rehabilitation programs in southern Sweden are valuable sources of long-term support. In times of financial constraints within the healthcare sector, such programs need resources to accommodate the growing number of older adults with long-term SCI. Well-coordinated follow-up routines at an SCI Unit will likely be financially rewarding in the long-term perspective.

### Associations between health-related quality of life and age, gender, and injury characteristics

Our findings indicate, not surprisingly, that the higher the level and the more complete the lesion, the more limitations in physical functioning and social interaction. In comparison, some previous studies have found a relationship between level and severity of injury and HRQoL [4, 6, 9], whereas others have not [7, 8]. A plausible explanation for this discrepancy may be the use of different assessment tools. Indeed, two studies using the SCI QL-23 [4, 6], both reported on significant relationships between the functioning domain of HRQoL and neurological impairment. Together with our results, these findings support the validity of the assessment tool in that subjective physical limitations conform with objective impairments [6].

### Associations between changes in health-related quality of life over 6 years and age, gender, and injury characteristics

In the multivariable analyses, a lower level and less severe SCI was associated with worse outcomes regarding depressive symptoms and global QoL over time. This might not be expected, yet, others have found similar results [27, 28]. In addition, we have previously found an association between more depressive symptoms, a lower satisfaction with somatic health and an AIS D injury in the SASCIS sample [19, 20]. The literature provides some explanation to these somewhat counterintuitive findings. For example, persons with less severe SCI can perceive a lack of understanding for their disability due to less visible impairments, and experience more pain and fatigue when walking [29]. The findings emphasize the need for increased attention to persons with AIS D injuries in the clinical setting. However, according to our clinical experience, persons with long-term AIS D injuries may have been excluded from follow-up programs some time after injury as their physical impairment seemed modest. The interval between their follow-ups may also have been prolonged as their potential SCI-related problems were regarded as possible to handle by primary health care providers. Given the many dimensions of mood and global QoL that can be affected after injury, also persons with less severe SCI need access to SCI-specialized rehabilitation professionals to mitigate negative changes over time.

Surprisingly, there was no relationship between changes in HRQoL domains covering SCI-related disability and sociodemographics and injury characteristics. One might expect that persons aging with more severe SCI would experience negative change in these domains as the aging process in itself entails declines in functioning. However, reaching older age with a disability may be perceived as a time of normalization as other people start to experience age-related impairments. Persons with a long-term disability may also be better prepared for age-related challenges as their resilience and adaptive skills have developed [30].

Given the broad construct of global QoL, the amount of variance explained by the independent variables (43%) can be considered rather high. However, no or little amount of variance was explained in the other HRQoL domains. These findings are actually encouraging as the investigated sociodemographics and injury characteristics are not modifiable by intervention. Further studies investigating modifiable factors related to changes in HRQoL are therefore needed to provide targets for interventions to support HRQoL in older adults aging with long-term SCI.

### Strengths and limitations

Several strengths of the SASCIS have been previously described, such as the use of internationally established assessment tools, the consistency of the data collection, limited missing data, and the representative study population [16]. The longitudinal design of the current study allows us to assess the effect of aging with long-term SCI on different domains of HRQoL over time. Furthermore, we used a validated questionnaire to assess HRQoL, developed specifically for people with SCI and for Swedish conditions. It is possible that healthier subjects from the original sample were recruited for the second assessment and that participants with poorer HRQoL were lost to follow-up. As data protection regulations prevent collection of data on the nonparticipants’ health status and QoL, it is not possible to eliminate such bias.

## Conclusions

Older adults aging with long-term SCI exhibit large variations in all HRQoL-domains and have the potential to maintain a relatively high and stable level of HRQoL over time. They show few signs of distress and depression and a high level of global QoL, despite some feelings of physical and social limitations and difficulties regarding injury-related problems. The associations indicate that persons with AIS D injuries may need increased attention in the clinical setting to mitigate negative changes in depressive symptoms and global QoL over time. This study can serve as a starting point for future research to identify modifiable factors associated with changes in HRQoL in older adults aging with long-term SCI.

## Data Availability

To attain confidentiality, all data were archived according to the Swedish Act concerning the Ethical Review of Research involving humans and are available from the corresponding author upon reasonable request.
